# Systems Challenges in Accessing Medicines among Children under Thailand’s Universal Health Coverage: A Qualitative Study of a Provincial Public Hospital Network

**DOI:** 10.3390/children9040552

**Published:** 2022-04-13

**Authors:** Rangsan Daojorn, Puckwipa Suwannaprom, Siritree Suttajit, Penkarn Kanjanarat, Prangtong Tiengket, Marc Lallemant

**Affiliations:** 1 Health and Medicine Policy Center (HMPC), Faculty of Pharmacy, Chiang Mai University, Chiang Mai 50000, Thailand; rsdj.dj@gmail.com (R.D.); siritree.s@cmu.ac.th (S.S.); penkarn.k@cmu.ac.th (P.K.); 2 Omkoi Hospital, Chiang Mai 50310, Thailand; 3 Public Health Promotion, Research and Training Foundation, Chiang Mai 50000, Thailand; prangthongg@gmail.com (P.T.); marclallemant@gmail.com (M.L.); 4 Programme for HIV Prevention and Treatment (PHPT)/IRD Unite, Chiang Mai 50000, Thailand

**Keywords:** accessibility, medicine, children, health system, medicine management

## Abstract

Lack of access to child-appropriate medicines results in off-label use. This study aimed to explore medicine management for paediatric patients and to highlight the challenges of the healthcare system under the universal health coverage of Thailand. Semi-structured interviews were conducted with 35 healthcare practitioners working in the public hospital network of Chiang Mai province from February to September 2020. Participants were asked about their experiences in managing the medicine supply for children. Findings revealed that paediatric patients had limited access to age-appropriate medicines. Children’s medicines are rarely selected for inclusion into hospital formularies because of constraining regulations and limited budgets. Additionally, child-appropriate formulations are unavailable on the market. Pharmaceutical compounding is unavoidable. Prepared products are provided weekly or monthly because of product stability concerns. Often, tablets are dispensed, and caregivers are instructed to cut up a tablet and disperse it in syrup in order to obtain a smaller dose in a dosage form appropriate for children to use at home. Without systematic support, access to safe and quality medicines for children is limited.

## 1. Introduction

Achieving universal health coverage (UHC) is a key target of the United Nations’ Sustainable Development Goal 3, which is to ensure healthy lives and promote well-being for all people of all ages [1]. Thailand is considered an international benchmark for the implementation of UHC, which focuses on improving access to health services, particularly primary healthcare [2,3,4]. Thailand’s Ministry of Public Health (MOPH) plays a key role in managing the public sector healthcare system, implementing its policies through its network of public hospitals [4,5]. After decades of developing health infrastructure and extending financial risk protection, the entire population of the country is covered by one of three public health insurance schemes: the Civil Servant Medical Benefit Scheme for government employees, the Social Security Scheme for private sector employees and the Universal Coverage Scheme for the general population [3,4]. Between 1996 and 2015, catastrophic spending fell from 6% to 2% [3], and the use of outpatient services increased among low-income, unemployed and chronically ill groups [6]. Many services are provided free of charge, including outpatient and inpatient services, high-cost care, accident and emergency services, disease prevention and health promotion services, community home care and rehabilitation services [4].

A referral system ensures a continuum of health services. Within the network, services ranging from primary care to specialised, tertiary care are organised to address the specific health problems of regional populations [7]. Small- and medium-sized district hospitals serve as first-level referral hospitals. Serious cases exceeding their capacity are transferred to an intermediate-level referral hospital, which may be a large district hospital in the network. The most critical cases are transferred to provincial, regional or teaching hospitals that are high-level referral hospitals [8].

Together with the organization of health services, personnel management, public information, financing and stewardship, access to medicines and health technologies are some of the essential elements of the healthcare system [9]. They are among the most cost-effective health interventions provided if they are available, affordable, of good quality and used rationally [10].

In Thailand, a National List of Essential Medicines (NLEM) has been established and is reviewed regularly based on health needs, safety, efficacy, cost-effectiveness, budget impact and affordability [11]. All medicines listed in the NLEM are included in the three public health insurance schemes’ reimbursement packages. Vital high-cost medicines for specific conditions are included in the list with strict indications for use and specific follow-up [12].

At the hospital level, a hospital committee, called the Pharmaceutical and Therapeutic Committee (PTC), is responsible for the inclusion of seleced of medicines into the hospital formulary [12,13]. The PTC ensures that medicine purchases comply with government regulations to guarantee reimbursement. According to these regulations, the proportion of essential medicines in the hospital’s formulary must be at least 70% and the proportion of hospital medicine expenditure on essential medicines be at least 60%, depending on the level of the hospital. Only one brand for each generic product is allowed and the prices of the medicines must be at or below the average price. Orphan and high-cost medicines are supplied centrally by the Government Pharmaceutical Organisation. Group purchasing and price negotiations are done at the regional or provincial level. As a result, more than 90% of the necessary essential medicines are available in public hospitals [13].

In the medicine selection process, the PTC has to balance the proportion of essential medicines against the budget, the service capacity and the regulations of the hospital. Therefore, being listed in the NLEM formally guarantees the availability of medicines in public hospitals. In the NLEM, essential medicines are indicated by their generic name and specific dosage form.

However, there are no specific provisions to address the specific medicine needs of children. In fact, there is no national list of essential medicines for children in Thailand, and there is a high incidence of off-label prescription and compounded preparations. Pharmaceutical compounding is defined as “a practice in which a licensed pharmacist, a licensed physician, or, in the case of an outsourcing facility, a person under the supervision of a licensed pharmacist, combines, mixes, or alters ingredients of a drug to create a medication tailored to the needs of an individual patient” [14]. These medicines are essential for patients with specific needs that do not have a licensed or commercial medicine available on the market [14,15]. However, compounded products are considered unlicensed medicines. The FDA does not verify the safety or effectiveness of these preparations. To better understand the situation, we conducted qualitative research to investigate the management of medicines for children within a network of provincial public hospitals where services are provided to people under the jurisdiction of the UHC. We used the Management Sciences for Health (MSH) framework, which identifies four functions of medicine management: selection, procurement, distribution, and use. These functions are supported by specific policies, laws and regulations [16].

## 2. Materials and Methods

### 2.1. Study Design

This qualitative research explored the management of children’s medicines as experienced by health practitioners working in the public hospital system of Chiang Mai province. The researcher, (R.D.), is a pharmacist working in the Chiang Mai public hospital system. The other team members, P.S., S.S. and P.K., are faculty members at Chiang Mai University. Their research focuses on access to health systems and rational use of medicines. They had previously established relationships with potential study participants. Qualitative research using content analysis as a theoretical framework was conducted using semi-structured, face-to-face interviews, accompanied by observations.

### 2.2. Study Context

The public hospital system in Chiang Mai province, Thailand, was used as a study site. Chiang Mai is the largest city in northern Thailand. In mid-2020, it had a population of 1,629,434, of which 304,489 were children under the age of 18 [17]. The public hospital system consists of 24 hospitals: 1 provincial hospital, 4 large district hospitals, 18 medium-sized district hospitals and 1 small district hospital that work together as a network of health services. In addition, there is a teaching hospital in Chiang Mai. Each hospital has its network of primary health centres at the sub-district level. In total, the number of health centres is 267 [17]. In these centres, services are provided by nurses and public health staff. At the district hospital level, a multidisciplinary team is available. In the provincial hospital, in addition to a full team of health staff, specialist doctors can treat complex health problems. The teaching hospital is fully equipped with technology and specialist staff to serve as a tertiary care unit. The higher the capacity of the hospital, the more comprehensive the hospital formulary is.

### 2.3. Selection of Participants

Potential participants were healthcare personnel involved in the medicine management in public hospitals of the Chiang Mai provincial health network. In order to obtain information from staff working at all levels in the hospitals, the sampling started with the selection of hospitals. As shown in Table 1, the teaching hospital, the provincial hospital and four large district hospitals were selected. In each hospital, the researcher contacted the head of the pharmacy department and presented the project and the research protocols. With their approval, we asked them to recommend the key people in the hospital responsible for managing the supply of medicines for children. Participants were then invited to take part in the study. Written informed consent was obtained before collecting the data. For medium-sized district and sub-district hospitals, the site was randomly selected. The researcher collected and analysed the data after visiting each site. The data reached saturation in 5 sites (out of 18) for the medium-sized hospitals. For the sub-district hospitals, saturation was reached in 10 sites. A total of 35 health personnel from 21 hospitals participated in this study. No one refused to participate in the study or dropped out.

### 2.4. Data Collection

The interview guide was developed based on the literature review and the MSH Pharmaceutical Management Systems Framework [16]. In this study, medicines were defined as prescription medicines in hospitals only. The questions in the interview guide were as follows:

- Please explain how children’s medicines are selected, procured, distributed and used in your hospital.
- Is there a system specifically designed for children’s medicines?
- Please describe the problems encountered in accessing or using medicines for children in the current system.
- Please specify what solutions to these problems you think are possible.

The interview guide was validated by three experts including two faculty members specialising in pharmaceutical systems management, and the head of a pharmacy department in a public hospital. A mock interview was conducted within the research team and a pilot test of the interview process was conducted. All interviews were conducted and field notes taken by the researcher (R.D.) at the participants’ workplace. The interview was audio recorded. At the end, the researcher summarised the key messages for the participants to validate.

Observations were made at the interview site to find out more about the preparation site and the process of preparing the child formulations. Photographs were taken. Other preparation-related documents were requested, including formulations for compounding and information on their shelf lives. Data collection took approximately 1 to 1.5 h per participant. Additional telephone interviews were conducted with 8 participants during data processing to clarify unclear points. Data were collected from February to September 2020.

### 2.5. Data Processing and Analysis

The data collected during the interviews was our main source of information, supported by the observations. The audio recordings of the interviews were transcribed verbatim, checked for completeness, and anonymised by the first author (R.D.). Data from the field notes and observations were incorporated into the transcripts. The first author generated the initial codes from the notes. Then two researchers (R.D. and P.S.) identified and organised themes and reflected on and discussed findings and interpretations. Disagreement was discussed until reaching consensus. Findings were presented to S.S. and P.K. for comments and feedback.

## 3. Results

A total of 35 healthcare personnel, including 9 department head pharmacists, 13 staff pharmacists, 10 nurses, two paediatricians and one general practitioner participated in this study. They represented perspectives of healthcare personnel involved in managing medicine access for paediatric patients of a teaching hospital, a provincial hospital, large district hospitals, medium district hospitals and sub-district hospitals. The results of this study are presented according to medicine management systems of selection, procurement, distribution and use.

### 3.1. Children’s Medicine Selection

According to the Ministry of Public Health (MOPH) regulations, the hospital’s PTC is responsible for medicine selection for the hospital formulary. This study found that the PTCs use generic drug selection criteria for any medicines, including medicines for paediatric patients. A single drug selection system is applied. Age-appropriate dosage forms for paediatric patients are limited in districts and sub-district hospitals. In larger hospitals, where the most complex cases are referred to specialist paediatricians, the list of child-appropriate formulations is larger than that in smaller hospitals with only general practitioners.

- **Medicine selection systems are the same across all categories, including paediatric medicines.**

All participants indicated that their hospitals use a single drug selection system where generic criteria are applied to any medicines. Medicine selection criteria include the assessed needs based on number of cases, existence of medicines available in the same therapeutic class already in the hospital formulary and overall ceiling of the proportion of and budget for non-essential medicines for public hospitals. As a result, medicines for children with age-appropriate dosage formulations available in the hospitals are for common illnesses, such as antibiotics (amoxicillin, erythromycin, and co-trimoxazole), antipyretics (paracetamol), antiemetics (domperidone), antispasmodics (hyoscine), and cough and cold medicines.

“The overall medicine management system for children does not differ from medicine for adults and no separate or special system exists.”

(FP4, staff pharmacist at a medium hospital)

- **A greater number of child-appropriate medicines are available at referral hospitals.**

In large district, provincial and tertiary care hospitals, more comprehensive lists with a greater variety of children’s medicines are available than those in smaller hospitals. In the larger hospitals, paediatricians provide specialised care for children. They play a key role in medicine selection, particularly child-appropriate medicines in the hospital formulary. Their roles included preparation of children’s medicine information for PTC review, negotiation with the PTC to acquire child-specific formulations based on needs, prescribing medicines and giving advice on medicine use to caregivers. These functions are significant parts that enhance the hospital’s capacity in caring for children who need more specialised care.

“For instance, we need levetiracetam oral solution. Previously in our hospital, only pills were available because the oral solution is very expensive. We have to explain why the oral solution is essential. The pill is too big for young children, as young as 1 month old, and is inconvenient to break and crush. It doesn’t work. Recently, they (the PTC) allowed us to have it (oral solution).”

(AD1, paediatrician at a provincial hospital)

### 3.2. Children’s Medicine Procurement

In acquiring medicines for public hospital use, drug procurement must conform to the government regulations to guarantee reimbursement. Although the need for child-appropriate medicines is presented, they might not be included in a hospital formulary because of limited budget. Even with the PTC approval, many child-appropriate formulations are unavailable in the Thai market. Therefore, medicine compounding is inevitable. In small hospitals with limited resources, compounded products are of doubtful quality due to stability concerns and dispensed only for a short period.

- **Government procurement regulations in purchasing essential medicines rule over the need for children’s medicines.**

Government regulations require public hospitals to procure at least 70% of their medicine items for essential medicines on the NLEM. Additional regulations limit the allowance of the medicine budget for non-essential medicines. When balancing demand, budgets and allowances for non-essential medicines and child-appropriate formulations are seldom selected for the hospital formulary.

“We have a limited budget and have to balance costs and benefits. The PTC board will discuss budget allocation. The problem is the fixed ratio of essential and nonessential medicines. We cannot have all that we want. The ratio must be kept as allowed.”

(MP5, pharmacy department head in a large district hospital)

- **Unavailable child-appropriate dosage forms on the market.**

In the case where the PTC agrees that child-appropriate formulations should be on the hospital list, there are several problems in acquiring those formulations. First, many products available on the market are inappropriate for children, e.g., phenobarbital elixir containing alcohol. Second, purchasing orders are too small because some products are infrequently used, although they are important for children. Third and most importantly, many child-appropriate preparations are simply unavailable on the market.

“We compound phenobarbital syrup for our paediatric patients because the formula sold in the market is in elixir form, and the percentage alcohol content is too high. So, doctors stopped using it.”

(MP1, staff pharmacist in a large district hospital)

“Do we have many patients? Is the drug important? When only 10 patients are admitted for one whole year, they might not let us open an account for this order. In this case, we will refer the patients to other hospitals stocking this medicine.”

(AP1, staff pharmacist at a provincial hospital)

- **Compounding preparations is unavoidable** **.**

As child-appropriate products are unavailable on the market or the hospital list, practitioners have to modify available products for their paediatric patients. Compounded medicines are prepared and dispensed. In many cases, medicines are given as adult pills or capsules, which caregivers are instructed to crush, divide into the required amounts, and mix with liquid before administering to their children. An education session for parents or caregivers is added to ensure that medicines are appropriately prepared at home.

“(In some cases, child-appropriate formulations are unavailable) so we teach the parents to crush a tablet, break a capsule, mix with water or juice, before giving it to their child. We don’t know if the drug dose is correct or not, but it’s better than nothing, better than the child not receiving medicine at all.”

(UP3, staff pharmacist at a teaching hospital)

- **Paediatric medicines are compounded with limited resources.**

Data from observations showed that compounded products for children are prepared in a limited area possessing available resources. Standardizing the compounding process is an issue. The compounded products are provided in a plastic bottle or a narrow-necked amber glass bottle. In some cases, a prepared suspension thickens over time resulting in difficulty of drawing and measuring medicines. Some formulations requiring special techniques to prepare, such as omeprazole, are only available in large hospitals.

Without hospital compounding formulas, pharmacists rely on recipes shared by larger and specialised hospitals. They use simple syrup and glycerine to prepare suspension, with no preservatives. Products are compounded using available resources. They are dispensed in small quantities with short shelf-lives (7–30 days), based on prior experience. Variations in medicine strength and indicated shelf-life are shown in Table 2.

### 3.3. Children’s Medicine Distribution

Compounded products for paediatric patients are prepared in small amounts due to their short shelf-life. Parents or caregivers must make frequent hospital visits to get a freshly prepared compounded product. In some hospitals, medicines are dispensed in tablets or capsules with instructions for preparing before use at home. Physicians and pharmacists are concerned about this issue but accept these limitations.

- **Short shelf-life products are burdensome for caregivers.**

With limited resources, pharmacists compound medicines for children, but these preparations have questionable stability. Concerning stability and safety, the compounded preparations are dispensed for only one week or one month of use. Parents or caregivers have to revisit the hospital weekly or monthly to obtain another bottle of medicine. The traveling cost is problematic for some patients. In many cases, pills or capsules are dispensed. Caregivers are responsible for compounding the medicines for their child before use.

“We use a compounded preparation for this medicine. The problem is that its shelf-life is only for one month. Parents who cannot come back to refill the medicine have to use pills and prepare them before use by crushing and mixing the powder with water or sweet drinks by themselves.”

(UP3, staff pharmacist at a teaching hospital)

- **With limited resources, having something is better than nothing.**

Physicians and pharmacists are concerned about compounded products’ stability, adherence, and acceptability. With limited resources and no system support, they are forced to accept these limitations and perform their best with what they have.

“We are unsure about the medicine’s stability. Even though the textbook states that it lasts one month, we must admit that the context is not exactly standardised as a production unit. Thus, we are comfortable with 7 days shelf-life.”

(FP6, staff pharmacist at a medium district hospital)

“If you ask me how confident I am, I’m not sure. However, the children must have the medicine for their condition. That is, we have to accept it. We have no choice.”

(AD1, paediatrician at a provincial hospital)

### 3.4. Children’s Medicine Use

Healthcare providers are concerned about prescribing medicines for their young patients. They are aware of their resource limitations. A supporting system among public hospitals has been developed. When dispensing medicines requiring home preparation, an education session with caregivers is being provided.

- **Prescribing medicines for paediatric patients remains a concern.**

Practitioners are concerned with prescribing medicines for children with any condition. In primary- and secondary-care settings, where nurses and general practitioners are the primary prescribers, they are more likely to treat only common conditions, and to refer children’s cases to a specialist at a larger hospital.

“I become a little tense when dispensing children’s medicines, where dosage must be well calculated. I can’t remember all. I have to open a manual or search the Internet.”

(SN1, nurse at a subdistrict hospital)

“If a case is too complex, exceeding the hospital potential, it must be sent to our network district hospital. Especially in children’s cases, patients will receive close monitoring by a specialised doctor and get specialised medications.”

(FP3, pharmacy department head at a medium hospital)

- **A supporting system within a hospital network is being developed.**

Within the province, a hospital network for cooperative health services is being developed. A consulting system is in place for a general practitioner or primary care staff seeking advice or discussing disease diagnosis and medication use. This system helps the generalists to ensure appropriate care and avoid case overload at a referral hospital. Within the network, pharmacists work cooperatively in supporting excipients, compounding recipes and sharing information for case management.

“We have a consulting system, and in some cases, we don’t have to refer every patient. In cases that they need medicines that we don’t have on our list, pharmacists, within the network, will coordinate and manage to obtain those medicines for their patients.”

(FP1, pharmacy department head at a medium hospital)

“We receive excipient for preparing oseltamivir suspension from our networking provincial hospital.”

(FP6, staff pharmacist at a medium district hospital)

- **Educating caregivers is one way to ensure the appropriate use of medicines.**

In the case where tablets and capsules are dispensed for paediatric patients, parents and caregivers are instructed on how to prepare the medicines for their children before use. Educating parents or caregivers on medicine preparation becomes essential. The efficacy and safety of these products can only be assessed by monitoring clinical outcomes of the patients on follow-up visits.

“We have to evaluate parents, first (for their readiness and competency). When they are ready, we dispense the pills for them, which is better for stability. However, we have to explain to them well.”

(MP4, staff pharmacist at a large district hospital)

“It constitutes a matter of trust. A physician must be sure that the parents can prepare and administer the medication to their child. We cannot follow them home. We can only assess them based on clinical outcomes at follow-up, that’s all.”

(AD1, paediatrician at a provincial hospital)

## 4. Discussion

This qualitative study exploring the perspectives of healthcare personnel involved in medicine management in Chiang Mai, Thailand demonstrated the complexity of managing children’s medicines from medicine selection by the hospital to its use by patients. The most prevalent issues in children’s medicines found in this study included lack of a specific selection system for paediatric medicines at the hospital level, the unavailability of some medicines with age-appropriate formations in the market, limited budget for medicines not in the NLEM list, some unstandardised medicines compounded in the hospitals, and insufficient follow-up on medicine use in children.

Thailand is an international example for implementing a universal health system focused on improving access to health services and medicines [2,3,4]. The backbone of this success is the national coverage of district hospital systems, resource sharing within the province, a regional referral system, and well-structured healthcare financing [2,3,4]. Regularly updated NLEM, pooled procurement and accompanying regulations help to ensure access to essential medicines under the UHC setting [11,18,19].

The UHC systems with the NLEM work effectively for ensuring access to essential medicines for most diseases. The UHC benefit package covers high-cost interventions and orphan medicines [4,11]. However, these research findings reveal several system challenges to accessing quality medicines for children. Medicines for children in age-appropriate dosage forms available in most public hospitals are those for common problems, such as antibiotics, antipyretics, anti-emetics, antispasmodics, and cough and cold medicines. Child-appropriate formulations for other conditions, such as heart problems, hypertension, and viral infections, are limited due to several factors [20]. In public hospitals, drug procurement must conform to the government regulations to guarantee reimbursement. The regulations require public hospitals to procure 70 to 100% of their medicine items as essential medicines, specifically no less than 70% for regional hospitals, 80% for provincial hospitals, 90% for district hospitals and 100% for sub-district hospitals [13]. When balancing among demand, budget and allowance for non-essential medicines, child-appropriate formulations are seldom selected. This study found that carbamazepine oral suspension is not on a hospital formulary because only its tablet dosage form is indicated on the NLEM. Acyclovir oral suspension is seldom found at a small hospital, even though it is listed on the NLEM. Compounding for hospital use is commonly found because of low volume demand and high price of the product.

Additionally, many child-appropriate preparations are unavailable on the market. Some oral solutions might be available elsewhere but not in Thailand, such as omeprazole, spironolactone, or furosemide. With these limitations, children’s medicines are often provided as compounded preparations using medicines for adults. In resource-limited hospitals, compounded products have doubtful stability and are dispensed for a short period. Tablets and capsules are often dispensed with instructions for home preparation. Practitioners are obliged to accept these limitations. Efficacy and safety of these products can only be assessed by monitoring the clinical outcomes of the patients on follow-up visits.

In Thailand, where only 10–20% of patients are pediatric patients [17], children’s medicines are not on the priority list in the medicine selection process. Manufacturers lack incentives to produce children’s medicines because of low demand, small profit margins and difficulties in storing child-friendly dosage forms [21,22]. Although medicine compounding is common practice [23,24,25], its potential negative consequences are well known, i.e., drug instability, inaccurate dosing, altered pharmacokinetics and bioavailability resulting in treatment inefficiency and impaired patient safety [23,26,27,28,29,30]. Therefore, certain standards are required [29,31,32], including a designated preparation station, standard formulas, proper equipment, trained personnel, and others. According to this study, such standards are rarely observed or mentioned. The situation is similar to a survey conducted in European hospitals, where little harmonization existed for formulations or information on the stability of the products [33]. A survey conducted in the Netherlands found that half of the compounding performed by nurses at paediatric wards was not fully concordant with the summary of product characteristics (SmPC) or the local hospital protocols [24].

In general, guidelines for assigning beyond-use-date, nonsterile compounded preparations are required to be packaged in tight, light-resistant containers and stored at controlled room temperature, and to be used within 14 days for formulations containing water [32]. Due to concerns about sub-optimal practices, medicines are dispensed for a shorter period, requiring parents and caregivers to visit the hospitals on a weekly basis, resulting in extra expense for the caregivers. Alternatively, dispensing pills or capsules for oral-liquid preparation at home is burdensome for parents or caregivers, and it requires clear instructions and training sessions [27,34].

The conditions of medicine management for paediatric patients in Thailand reflect a complex situation of medicine access for children. A similar issue occurs globally [35,36,37,38,39,40]. At the global level, regulators, i.e., the FDA, EMA, and the WHO have developed prequalification systems to encourage the industry to improve paediatric formulations and facilitate the production of generic paediatric products by the industry [21,41,42,43]. In the U.S. and EU countries, major initiatives have focused on stimulating research into children’s medicines driven by drug regulatory reforms [35,36,38]. In Australia, a paediatric medicines advisory group has been established and a national paediatric dosing reference has been established [35]. In Switzerland, an on-line database for national dosage recommendations for children based on the latest available scientific evidence and best clinical practice has been developed [44]. In Tanzania and Ireland, national guidelines for medicine compounding in hospitals have been established [45,46]. These initiatives provide supporting structures for children’s access to medicines in their counties.

The findings from this study call for the development of specific medicine management systems for children at national and local levels. To improve children’s access to medicines, a systematic approach focusing on the interconnections between system components: national policies and regulations, drug management systems, health financing, human resources, health information and service delivery should be considered [16,47]. The establishment of a NLEM for children (NLEM-c) [43] would increase the chance of medicine coverage under the Thailand UHC and might lead to improved availability of children’s medicine in hospitals. Special procurement initiatives and processes should be implemented to ensure access to high cost and orphan medicines [11]. National guidelines for pharmaceutical compounding should be developed and implemented for endorsing standards of medicine compounding in all hospitals. Standard formulations will help in harmonizing the strength and stability of compounding products. Education and training on medicine compounding is recommended [31,32,45,46]. A platform for sharing compounding formulations and stability information among practitioners is essential [48,49]. Additionally, standardised education and training for parents and caregivers for children’s medicine preparation is crucial [34].

To the best of our knowledge, this constitutes one of the very few qualitative studies to provide a comprehensive picture of constraints in medicine management systems at hospitals at the provincial level that affect access to children’s medicines. However, this study encountered some limitations. The study included informants only from public hospitals in one province in Thailand. However, all types of hospitals were included. Given Thailand’s healthcare context and procedures developed within the selected provincial hospital networks, the interpretation of the study findings must be carried out with caution. Other limitations should be addressed here, e.g., the researcher (R.D.) is a pharmacist in the medicine management system at provincial and hospital level and his prior knowledge and biases might affect the interpretation of the findings. As the research was conducted at public hospitals, situations might be inapplicable to private facilities. Nevertheless, we hope this study will provide useful information to better understand the interconnection between medicines and healthcare systems and that system structures are essential to improve access to medicines for children. Future research should explore the experiences of parents or caregivers in managing children’s medicines, initiatives to support standardised compounding recipes for children’s medicines and the development policies for increased access to child-appropriate medicines.

## 5. Conclusions

Access to child-appropriate medicines is limited, even in a country with a well-structured universal public health system. Limited budgets, lack of practice standards, inadequately trained personnel, absence of supporting policies and structures are intertwined barriers. To improve the access, national and local support systems are needed.

## Figures and Tables

**Table 1:** Study participants.

Hospital Type	Number of Hospitals in the Province	Number of Hospitals Selected	Number of Participants
Teaching hospital (1400 beds)	1	1	3
Provincial hospital (600 beds)	1	1	5
Large district hospital (90–200 beds)	4	4	9
Medium district hospital (30–90 beds)	18	5	8
Small district hospital (10 beds)	1	-	-
Sub-district hospital (OP case only)	267	10	10

**Table 2:** Selected examples of children’s medicines compounded in participating hospitals.

Medicine	Dosage Form Prepared	Strength (mg/ mL)	Indicated Shelf-Life Stability at Room Temperature (Day)			
			Teaching Hospital	Provincial Hospital	Large District Hospital	Medium District Hospital
Acetazolamide	Suspension ^1^	10, 50	10	7	-	-
Acyclovir	Suspension ^1^	20, 30, 40, 70, 80, 120	7	14	10	-
Aminophylline	Suspension	2	14	10	10	-
Atenolol	Suspension	2	30	21	-	-
Carbamazepine	Suspension	20	30	30	-	-
Chloroquine phosphate	Suspension	15, 20	-	30	7	-
Ciprofloxacin	Suspension	50	30	30	7	-
Hydrochlorothiazide	Suspension ^1^	1, 2, 5, 10	30	30	7	-
Isoniazid	Suspension	50	14	30	30	-
Omeprazole	Suspension	2, 5	15	30	14	-
Oseltamivir	Suspension	5, 10, 15	10	10	10	7
Phenobarbital	Suspension ^1^	3, 4, 5, 10	60	30	10	-
Prednisolone	Suspension ^1^	1, 5	30	7	7	7

^1^ In some hospitals, pills were dispensed with instructions for home preparation.

## Data Availability

The data presented in this study are a part of Master’s Thesis, approved by the Faculty of Pharmacy, Chiang Mai University.
